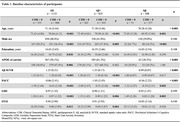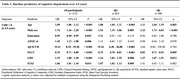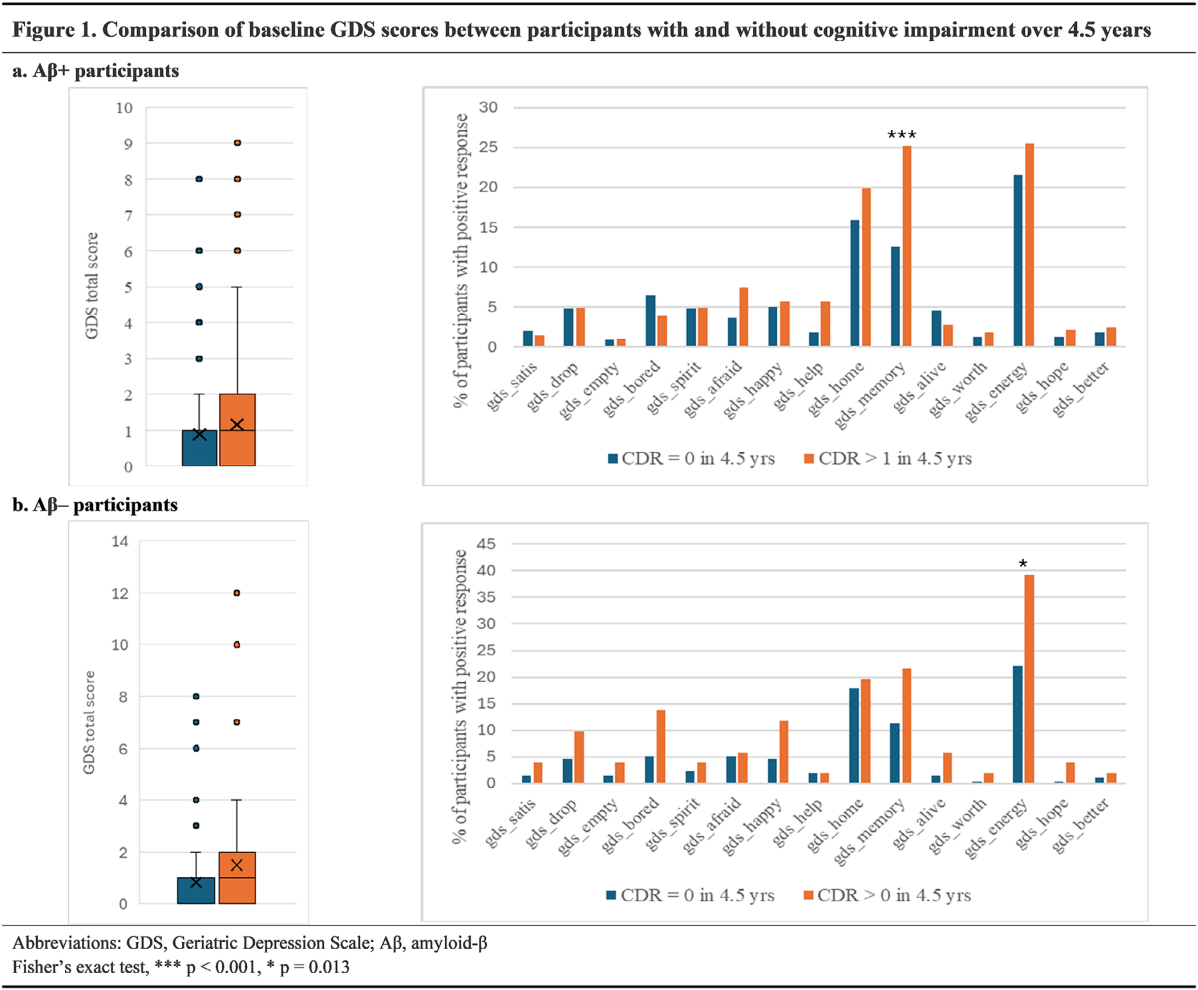# Association of baseline risk factors with subsequent cognitive decline in individuals with and without elevated brain amyloid enrolled in a preclinical AD treatment study

**DOI:** 10.1002/alz70857_107735

**Published:** 2025-12-25

**Authors:** Therese H Kim, David L Sultzer

**Affiliations:** ^1^ UC Irvine Institute for Memory Impairments and Neurological Disorders (UCI MIND), Irvine, CA, USA; ^2^ The UC Irvine Institute for Memory Impairments and Neurological Disorders, Irvine, CA, USA; ^3^ School of Medicine, University of California, Irvine, Irvine, CA, USA

## Abstract

**Background:**

This study examines the associations between baseline risk factors and future cognitive decline in individuals with and without elevated brain amyloid. Furthermore, the study investigates whether predictors of cognitive impairment differ based on amyloid status.

**Method:**

Among 1144 Participants from the A4 Study who attended the 4.5‐year visit (visit 66), 1133 with available Clinical Dementia Rating (CDR) data were included in the analysis. Baseline predictors assessed at visit 1 included age, sex, education, APOE genotype, amyloid‐β (Aβ) standard uptake value ratio (SUVR), Preclinical Alzheimer's Cognitive Composite (PACC) score, Geriatric Depression Scale (GDS) score, and State‐Trait Anxiety Inventory (STAI) score. Logistic regression models were used to evaluate the impact of these predictors on future cognitive impairment (CDR>0 at visit 66) in Aβ‐positive (Aβ+) and Aβ‐negative (Aβ−) participants. Additionally, baseline GDS total and item scores were compared between those who developed cognitive impairment and those who remained cognitively unimpaired.

**Result:**

Among 1133 participants (825 Aβ+ and 308 Aβ−), 282 (34.2%) Aβ+ and 51 (16.6%) Aβ− participants developed cognitive impairment over 4.5 years. Common predictors of cognitive impairment in both Aβ+ and Aβ− groups included older age, male sex, and lower PACC scores. Aβ SUVR was the strongest predictor of cognitive impairment in Aβ+ individuals, increasing the risk 15‐fold (OR=15.34, *p* <0.001), while its effect was not statistically significant in Aβ− participants (OR=3.04, *p* = 0.703). Similarly, baseline STAI scores were significantly associated with cognitive decline in the Aβ+ group (OR=1.07, *p* = 0.005) but not in the Aβ‐ group (OR=1.01, *p* = 0.882). Higher baseline GDS scores were significantly associated with cognitive impairment in both groups (Aβ+: OR=1.16, *p* =  0.008; Aβ‐: OR=1.27, *p* = 0.012). However, item‐level analysis revealed distinct patterns: in Aβ+ individuals, memory concerns showed the strongest association with future cognitive decline (*p* <0.001), whereas in Aβ− individuals, low energy levels were the most predictive symptom (*p* = 0.013).

**Conclusion:**

While common risk factors are associated with cognitive impairment regardless of amyloid status, some predictors and their presentations vary between Aβ+ and Aβ− individuals, suggesting distinct underlying mechanisms of cognitive decline.